# Innovative Care for Inflammatory Bowel Disease Patients during the COVID-19 Pandemic: Use of Bedside Intestinal Ultrasound to Optimize Management

**DOI:** 10.1093/jcag/gwac006

**Published:** 2022-03-10

**Authors:** Cathy Lu, Christopher Ma, Richard J M Ingram, Melissa Chan, Hengameh Kheirkhahrahimabadi, Marie-Louise Martin, Cynthia H Seow, Gilaad G Kaplan, Joan Heatherington, Shane M Devlin, Remo Panaccione, Kerri L Novak

**Affiliations:** Department of Medicine, Division of Gastroenterology and Hepatology, University of Calgary, Alberta, Canada; Department of Medicine, Division of Gastroenterology and Hepatology, University of Calgary, Alberta, Canada; Department of Community Health Sciences, University of Calgary, Alberta, Canada; Department of Medicine, Division of Gastroenterology and Hepatology, University of Calgary, Alberta, Canada; Department of Medicine, Division of Gastroenterology and Hepatology, University of Calgary, Alberta, Canada; Department of Medicine, Division of Gastroenterology and Hepatology, University of Calgary, Alberta, Canada; Department of Medicine, Division of Rheumatology, University of Calgary, Alberta, Canada; Department of Medicine, Division of Gastroenterology and Hepatology, University of Calgary, Alberta, Canada; Department of Medicine, Division of Gastroenterology and Hepatology, University of Calgary, Alberta, Canada; Department of Community Health Sciences, University of Calgary, Alberta, Canada; Department of Medicine, Division of Gastroenterology and Hepatology, University of Calgary, Alberta, Canada; Department of Community Health Sciences, University of Calgary, Alberta, Canada; Department of Medicine, Division of Gastroenterology and Hepatology, University of Calgary, Alberta, Canada; Department of Medicine, Division of Gastroenterology and Hepatology, University of Calgary, Alberta, Canada; Department of Medicine, Division of Gastroenterology and Hepatology, University of Calgary, Alberta, Canada; Department of Medicine, Division of Gastroenterology and Hepatology, University of Calgary, Alberta, Canada

**Keywords:** COVID-19, Coronavirus, Crohn’s disease, Inflammatory bowel disease, Ulcerative colitis, Ultrasound

## Abstract

**Background:**

The COVID-19 pandemic caused by SARS-CoV-2 has reduced access to endoscopy and imaging. Safe alternatives, available at the bedside, are needed for accurate, non-invasive strategies to evaluate disease activity. The aim of this study is to establish the impact of clinic-based bedside intestinal ultrasound (IUS) on decision making, reduction in reliance on endoscopy and short-term healthcare utilization.

**Methods:**

We conducted a prospective observational evaluation during the COVID-19 pandemic, of the impact of a regional comprehensive care pathway to manage IBD patients consecutively recruited with acute symptoms, or suspected new diagnosis of IBD. Clinic-based access to sigmoidoscopy and bedside intestinal ultrasound were evaluated, used to direct clinical care and avoid hospitalization or hospital-based endoscopy.

**Results:**

A total of 72 patients were seen between March 15 and June 30, 2020. Of these, 57% (41/72) were female, 64% had Crohn’s disease (46/72) with 14% (10/72) presenting with symptoms requiring investigation, of which 5 new cases of IBD were identified (50%). Immediate access to ultrasound and sigmoidoscopy led to meaningful changes in management in 80.5% (58/72) of patients. Active inflammation was detected by IUS alone (72.5%, 29/40) or in combination with in-clinic sigmoidoscopy (78%, 18/23) or sigmoidoscopy alone (78% 7/9). Six patients were referred to colorectal surgery for urgent surgical intervention including two patients admitted directly.

**Conclusion:**

Implementation of IUS as part of a clinical care pathway during the COVID-19 pandemic is a useful strategy to enhance care delivery and improve clinical decisions, while sparing other important acute care resources.

## Teaser

A centralized clinical care pathway integrating intestinal ultrasound in the management of acutely flaring inflammatory bowel disease patients avoids unnecessary emergency department visits and facilitates timely appropriate treatment during the COVID-19 pandemic.

## INTRODUCTION

The coronavirus disease 2019 (COVID-19) pandemic caused by the novel severe acute respiratory syndrome coronavirus 2 (SARS-CoV-2) has infected ~173 million patients worldwide and caused over 2.7 million deaths (1). In North America and Europe, the first wave of COVID-19 occurred in the spring of 2020, with the second wave emerging in the fall of 2020. In response, public health implemented drastic measures aimed at maximizing physical distancing to reduce viral transmission (2). As a result, substantial changes to the delivery of healthcare services followed such as shifting ambulatory clinic visits to virtual delivery (3). In addition, early actions in the pandemic limited access to many acute care resources such as endoscopy and hospital-based cross-sectional imaging. This may significantly impact the care of patients with inflammatory bowel disease (IBD) who may be flaring and require objective assessment to confirm disease activity prior to altering course of management.

Endoscopic visualization has historically been the gold standard for confirming luminal inflammation. However, the availability of routine endoscopy may be restricted as many healthcare systems divert resources to managing the pandemic. National and international recommendations suggested restricting routine endoscopic evaluation, limiting invasive testing to those carefully screened for influenza-like illness (ILI) and emphasizing use of often limited personal protective equipment (PPE) for these procedures (4–6). Consequently, non-invasive methods other than endoscopy to accurately evaluate disease activity during the COVID-19 pandemic are needed (6).

Intestinal ultrasound (IUS) at the point-of-care is an effective, accurate, non-invasive tool used to transmurally characterize IBD activity, objectively map disease extent and severity and exclude complications common to IBD patients such as strictures or fistulae (7,8). IUS performed by gastroenterologists has been increasingly adopted in IBD expert centres globally as a core component of patient-centred monitoring. IUS aids in real-time clinical decision-making, particularly for patients with established IBD and increasing symptoms (9). Clear evidence of inflammation on IUS can be used to support treatment escalation, while conversely, a normal IUS combined with serological and stool studies, suggesting absence of significant inflammation, allowing for reassurance and obviating the need for invasive endoscopy.

Here, we summarize the experience of using integrated IUS in a centralized IBD clinical care pathway to improve the efficiency and quality of care for acutely flaring IBD patients during the COVID-19 pandemic.

## MATERIALS AND METHODS

### Study Population and Design

We conducted a prospective, observational cohort study at The University of Calgary IBD Clinic (Calgary, Canada), a tertiary care referral centre servicing a population of ~1.2 million people in a large metropolitan area and multiple surrounding rural communities.

At the start of the COVID-19 pandemic, a centralized model was created for the Calgary zone: referrals for patients with established IBD with flaring symptoms, or patients with laboratory findings or clinical history highly suspicious for a new diagnosis of IBD were facilitated and expedited (seen within 7 days). The aim of this centralized model was to streamline care, optimize use of PPE, and provide consistent, safe, and timely access to care in an outpatient setting regardless of hospital affiliation/physician attachment. Long-term goals included avoiding visits to the emergency department or hospitalization and minimizing the need for patients to undergo acute care, hospital-based endoscopic evaluation.

The IBD flare clinic is a city-wide service allowing community-based gastroenterologists to refer their patients with IBD for rapid assessment in a centralized location. Referrals to the central care pathway were seen face-to-face in an outpatient clinic after being cleared for ILI symptoms. All patients were evaluated by one of two IBD-focused physicians (K.L.N. and C.L. in Calgary). Patients were screened for gastrointestinal infections (*Clostridoides difficile*), fecal calprotectin was recorded where available, in addition to routine laboratory investigations (C reactive protein/CRP, complete blood count and albumin).

Patients seen in the centralized flare clinic during the first wave of the pandemic (from March 15 to June 30, 2020) were followed through until December 31, 2020. Outcomes assessed included emergency department visitation, hospitalization and planned or unplanned surgery, and COVID-19 infections.

### Intestinal Ultrasound

IUS was performed by two experienced gastroenterologists (K.L.N. and C.L.) with training in IUS. IUS was performed using a multifrequency convex (3–10 MHz) transducer with a Samsung RS80A machine (Samsung Medison Co. Ltd, Seoul, Korea) or 2–9 MHz transducer with a Philips Epiq 7 machine (Philips Healthcare, Bothell, WA). All bowel segments were assessed in short and long axes. Abnormal bowel loops were captured as images to show bowel wall thickness, length of affected bowel, inflammatory fat and amount of hyperemia as measured by colour Doppler imaging. Stricture was defined as a thickened segment with a fixed narrowed lumen with or without prestenotic dilation, and with or without dysfunctional peristalsis. Additional penetrating complications were documented, characterized by disruption of the usual bowel wall patterns, associated extraluminal areas and variable hypoechogenicities with inflammation and fluid collections outside the lumen (10).

### Sigmoidoscopy

Where distal disease was known or highly suspected (established ulcerative colitis and new onset hematochezia), an outpatient, non-sedated flexible sigmoidoscopy was performed after informed consent was obtained (standard of care) in the outpatient clinic. Conventional adult gastroscope or pediatric colonoscopes were used (Pentax EG34-i10 and EC-3470LK). Biopsies were collected for histopathology.

### Analysis

Descriptive statistics from the first 72 patients evaluated through this pathway are presented. Age at diagnosis, Montreal classification, smoking status, CRP and fecal calprotectin were recorded for all patients (where data available).

The primary outcome was acute care hospital-based endoscopy avoidance. Secondary aims including impact on clinical decisions, defined as any substantive change in current medications such as biologic dose escalation/dosing interval increase/optimization, drug or class switch, medication addition (immune suppressant addition, corticosteroids, antibiotics etc.) and referral to colorectal surgery. Additional secondary aims included proportion of patients with objective evidence of disease activity detected by IUS or sigmoidoscopy or both, proportion of patients to the emergency department presenting and hospitalizations and testing for COVID-19 during the study period.

## ETHICAL CONSIDERATIONS

This study was approved and reviewed by the Conjoint Research Ethics Board at the University of Calgary. Each subject provided signed informed consent, which included use of de-identified images for research/publication purposes.

## RESULTS

We evaluated 72 patients in the centralized IBD flare outpatient clinic. Patient demographics and presenting details are summarized in Table 1. Most patients were female (41/72, 56.9%) with a median age of 40 years (range 20–85). The indication for evaluation was flaring Crohn’s disease (CD) (46/72, 64%), ulcerative colitis (UC) (16/72, 22.2%), or evaluation of symptomatic undiagnosed high-risk patients (10/72, 13.8%). Of the 10 symptomatic patients, 5 patients were confirmed to have a new diagnosis of IBD.

**Table 1.** Patient demographics of IBD flare patients attending clinic

Characteristics (%)	Calgary *n* = 72 (%)
Median age years [Range]	41 [27–60]
Female	41 (56.9)
Smoker	
Current	7 (9.7)
Non-smoker (includes previous smokers)	65 (90.3)
Crohn’s disease	46(64)
Age at diagnosis	
A1	1 (2.1)
A2	37 (51.3)
A3	8 (17.0)
Unknown	0 (0)
Disease location	
L1	18 (38.3)
L2	16 (34.7)
L3	12 (25.6)
Disease behaviour	
B1	20 (43.4)
B2	16 (34.0)
B3	10 (21.2)
Perianal disease with B1/B2/B3	8 (17.0)
Ulcerative colitis	16 (22.2)
Pancolitis	4 (25)
Left sided	9 (56.2)
Proctitis	3 (18.8)
Symptoms (no diagnosis)	10 (13.8)
New IBD	5 (50)
Medications at assessment	
Corticosteroids	7 (6.9)
Immunomodulatoralone∗	2 (2.8)
Anti-TNF†	17 (23.6)
Ustekinumab	5 (6.9)
Vedolizumab	10 (13.9)
Tofacitinib	3 (4.2)
5-ASA	6 (8.3)
Clinical trial‡	1 (1.4)
Combination Biologic§ and Tofacitinib	1 (1.4)
Immunomodulator and Biologic	4 (5.6)
Corticosteroid and Biologic or Immunomodulator	2 (2.8)
Corticosteroid and 5-ASA	1 (1.4)
Corticosteroid, 5-ASA and Immunomodulator	0 (0)
5-ASA and Immunomodulator	0 (0)
None	26 (36.1)
CRP	
Median CRP for patients with Crohn’s [Range]	4.1 [0.3 – 82]
Median CRP for patients with UC [Range]	3.9 [0.6 – 19.7]
Median CRP for patients with symptoms [Range]	20 [0.6 - 68]

∗Azathioprine or methotrexate.

Includes biosimilar CPT-13, originator infliximab, adalimumab and golimumab.

Clinical trial drugs included rizankizumab and upacitanib.

Biologic includes Vedolizumab, Ustekinemab and Anti-TNF.

**Figure 1.**
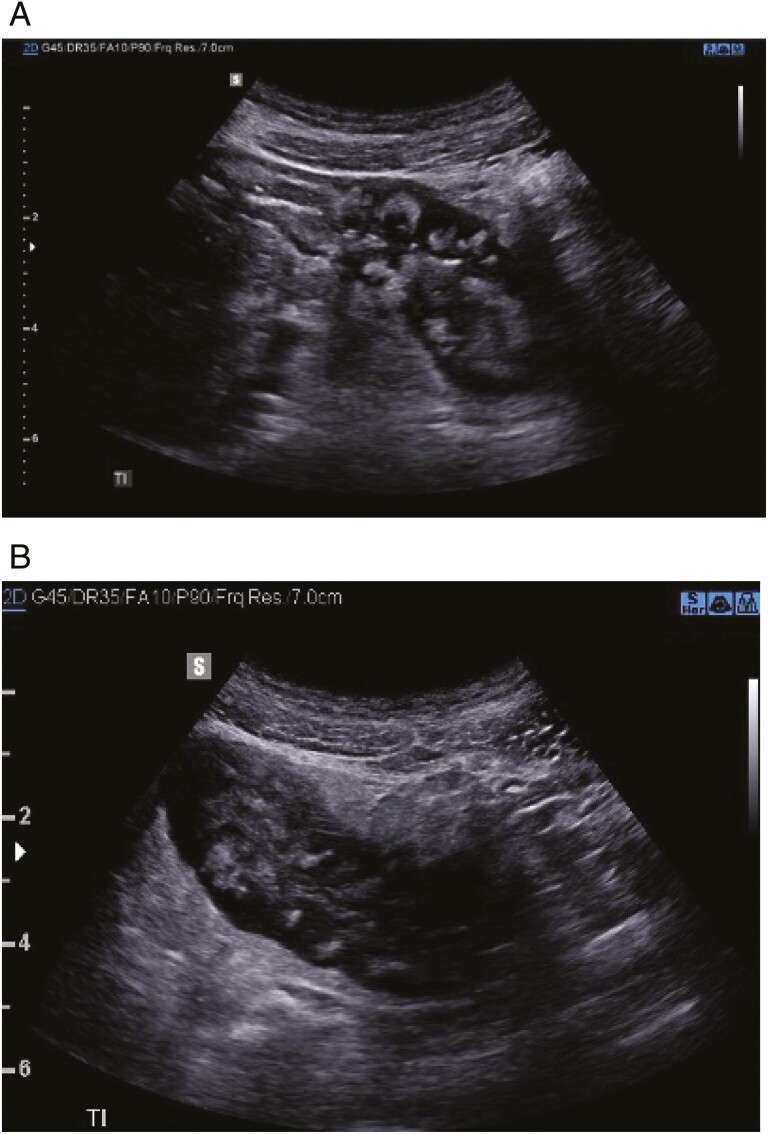
Illustrative intestinal ultrasound results showing high-risk, long-segment stricture in patient with ileal Crohn’s disease, necessitating change in management plan. A young man with abdominal pain and obstipation. Colonoscopy revealed stricture in the distal ileum with biopsy demonstrating chronic severe changes. Presented with increasing symptoms of chronic obstruction to the University of Calgary IBD clinic in April 2020. (A) demonstrates long- (>10 cm) segment stricture, severe wall thickening (yellow line, 1 cm) and wall apposition (white arrow demonstrates lumen). High-risk features include extensive loss of normal echostratification (wall layers with areas that are black/anechoic) (B), loss of normal motility with proximal dilatation.

A total of 63/72 (87.5%) patients were evaluated with bedside IUS. The majority of patients (55.5%, 40/72) had objective evaluation with IUS alone, with 31.9% (23/72) of all IUS conducted in combination with sigmoidoscopy (Table 2). The majority of patients evaluated with IUS exhibited active inflammation (47/63 75%) characterized by increased bowel wall thickness (BWT) with median maximum BWT of 6.0 mm (2.0–10.0 mm) and a median CRP of 5.7 mg/L (0.3–82). There were only eight available FC measures with a median of 520.5 µg/g (18–3700). There were eight strictures identified. Nine patients (12.5%, 9/72) underwent in-clinic sigmoidoscopy alone for known rectal disease or distal symptoms, with 7/9 (78%) exhibiting active inflammation. Two patients were not considered candidates for IUS due to obesity and complex pelvic anatomy, respectively. Almost all patients were successfully managed in the outpatient setting: two patients required direct hospital admission for ileocecal resection due to ongoing symptomatic obstruction and severe, high-grade stricture identified on bedside IUS (Figure 1). Acute care in-hospital endoscopy was avoided in the majority (80.6%, 58/72) of cases, with non-urgent subsequent colonoscopic examinations scheduled electively in the minority (8/72, 11.1%), given either suspicion of mild or absence of disease, needing routine confirmation. No patients were identified as having an enteric infection.

**Table 2.** Clinical decisions and outcomes

	Completion of intestinal ultrasound with or without flexible sigmoidoscopy (*n* = 72)		
	US only *n* = 40 (%)	Sig only *n* = 9 (%)	Sig and US *n* = 23 (%)
Activity/Inflammation present	29 (72.5)	7 (78)	18 (78)
Median Maximum BWT (active) (mm) [range]	6.0 [2–10]	n/a	5.2 [3.3–10]
Stricture	8 (20)	0	0
Avoided all acute care endoscopy	28 (70)	9 (100)	21 (91)
Further investigation∗	16 (40)	5 (55)	4 (17)
Non-urgent endoscopy	6 (15)	0 (0)	1 (4.3)
Change in clinical management†	32 (80)	7 (78)	19 (83)
Change in medication			
A) Corticosteroid start	5 (2.5)	1 (11)	7 (30.4)
B) Biologic Start/Optimize/Switch	12 (30)	1 (11)	5 (21.7)
C) Start/Optimize JAK	0 (0)	0 (0)	1 (4.3)
D) De-escalation	1 (2.5)	0 (0)	0 (0)
E) Surgical consultation	5 (12.5)	0 (0)	1 (4.3)
F) Other‡	17 (42.5)	3 (33.3)	8 (26)

n/a, not applicable.

∗Further investigation includes fecal calprotectin, stool infection studies including Clostridium difficile, further cross-sectional imaging such as computed tomography or magnetic resonance enterography.

US, Intestinal ultrasound; Sig, Flexible sigmoidoscopy. May have concurrent multiple changes in clinical management.

Other: includes rectal therapy, introduction of osmotic laxative, psyllium, loperamide, cholestyramine or amitriptyline.

Most patients, 84.7% (61/72), had a substantial management change in response to the detection of active inflammation by either IUS alone (80%, 32/40) or in combination with in-clinic sigmoidoscopy (82.6%, 19/23). Of these, 5/61 (8.2%) exhibited biologic dose escalation, 12/61 (19.7%) had either introduction of a biologic or a class switch, whereas 8/61 (13%) required topical or systemic corticosteroids. Six patients were referred to colorectal surgery for resection of complicated disease (all exhibited strictures, one with stricture and enteroenteric fistula identified) characterized by IUS, including two emergent surgeries given progressive, symptomatic obstruction. One patient was scheduled for colorectal surgery consultation and based on IUS findings, with no evidence of significant structuring, medical therapy alone (biologic class switch) was recommended.

COVID testing during the study period was restricted, unavailable to the patients at assessment in the flare clinic. All were screened for ILI symptoms. COVID testing was completed in 43% (31/72) of patients who attended the centralized flare clinic at least once (often more than once) in the subsequent time period, with only one patient testing positive, 5 months after evaluation. This patient recovered without need for acute care. There were six presentations to the emergency department/urgent care for gastrointestinal concerns: 4/6 were in patients with ongoing abdominal pain, established irritable bowel syndrome seen in flare clinic given prior ED/UC presentations, whereas two exhibited progressive complex penetrating CD who had been referred to colorectal surgery, requiring hospital admission. There were six additional admissions to hospital, of these two were for planned IBD-related surgeries, whereas four were unrelated (pneumonia, renal colic, esophagitis and esophageal cancer diagnosis).

## DISCUSSION

The COVID-19 pandemic has significantly impacted routine care delivery for patients with IBD. Virtual/telemedicine interactions have been prioritized, to best adhere to public health recommendations of physical distancing. Virtual care is an important innovation in care with clear benefit for patients with stable disease, well known to the provider; however, for those who have active symptoms, more comprehensive evaluation is essential to guide management decisions. This commonly involves endoscopic evaluation and cross-sectional imaging. Unfortunately, access to these resources has likewise often been limited during the pandemic. The integration of IUS in a clinical care pathway may offer strategic advantages for IBD management during this pandemic, including (a) expediting early, appropriate treatment decisions (medication intensification/surgical referral in patients with active disease vs. avoiding overtreatment of symptoms alone in patients without disease activity on IUS); (b) avoidance of unnecessary emergency department or hospital visits by facilitating comprehensive, objective outpatient assessment; and (c) deferral of acute care endoscopy by non-invasively and accurately staging disease activity.

IUS is increasingly employed as an accurate non-invasive tool to assess disease activity in both Crohn’s disease and ulcerative colitis (11,12). Mounting evidence supports accurate depiction of inflammatory activity, complications and post-operative recurrence detectable by IUS, comparable to MR or CT, yet is more accessible to patients in clinic (7,11–13). Evidence also highlights patient choice: ultrasound is consistently preferred over MR and endoscopy, for monitoring and investigation of symptoms (14). Despite high accuracy, several barriers have prevented IUS from being routinely implemented in clinical practice in North America. Recent evidence suggests physicians in the United States prefer to use radiation-based CT scan more often, particularly in the context of IBD flares (15). Performance and interpretation of IUS require specialized training and expertise. A significant paradigm shift in monitoring strategies towards more use of non-invasive, safe imaging performed by IBD-focused physicians is important, to facilitate its implementation. Provision of bedside IUS is recognized as an important patient-centred innovation for routine IBD care and practice change is therefore important.

Important exclusions when assessing active symptoms in IBD include infectious etiologies, namely, *Clostridoides difficile* and other gram-negative pathogens, in addition to the potential need to exclude COVID-19 as a driver. Isolated gastrointestinal symptoms as a single presenting complaint of COVID-19 infection are rare and rather occur more frequently in combination with other symptoms such as fever, cough and sore throat (16). Importantly, all patients were pre-screened for GI infections in addition to any ILI symptoms including fever, as a means to divert face-to-face assessment. During the first wave of the pandemic, access to routine COVID-19 testing was systematically limited to those with ILI symptoms in the region. Furthermore, none of the patients included presented with positive COVID tests in the study period (one positive in follow up). Bedside IUS was feasible to perform during outpatient clinic visits while adhering to PPE requirements. IUS is not considered an aerosol generating procedure; this pathway also facilitated the preservation of N95 respirators for use in other departments.

The additional recognized benefit of using objective evidence to guide treatment strategies as opposed to symptoms alone is in the avoidance of unnecessary, potentially harmful immune suppressants, such as use of corticosteroids that are not indicated. Chronic symptom may not be driven by inflammation, rather common conditions such as irritable bowel syndrome (IBS) or other functional etiologies. In this study, we demonstrate important contribution of IUS in reassuring the provider and patients regarding the absence of inflammation driving symptoms, as 42.5% of patients were treated with non-immune–based medical therapies such as cholestyramine or amitriptyline, effective for symptom management in conditions such as irritable bowel syndrome overlap.

There are some limitations to this observational study. Firstly, the IUS providers were not blinded to the clinical history or symptoms and are potentially biased towards favouring use of the tool to guide clinical decisions. This may result in greater impact seen for IUS in this cohort. However, it is part of routine, standard of care in this centre, as part of the comprehensive evaluation in addition to other objective monitoring tools. Secondly, the lack of available COVID testing at assessment may have missed possible concomitant COVID-driven gastrointestinal symptoms; however, no positive test related to the flare-clinic visit was identified with follow-up extended to the end of the year. This observational study took place early in the pandemic, when confidence in the safety of virtual care for ill patients with IBD was limited, opting preferentially for use of appropriate PPE and face to face assessment facilitating objective monitoring. It would have added strength to this evaluation to compare the clinic-based IUS and sigmoidoscopy assessment with those assessed virtually. This hypothesis generating observation provides impetus for future comparisons.

## CONCLUSION

The COVID-19 pandemic has challenged healthcare systems to alter ways in which care is delivered, while preserving patient outcomes. We were able to demonstrate that the use of clinic-based IUS provides an important patient-centred tool to facilitate the management of acutely flaring IBD patients while sparing ER and hospital visits. These findings provide an argument to advocate for the expansion of the role of IUS in the management of IBD during and beyond the COVID-19 pandemic. Successful integration of IUS into clinical pathways may also inform future optimal strategies for IBD care delivery post-pandemic.
